# A Single-Shot Non-Line-of-Sight Range-Finder

**DOI:** 10.3390/s19214820

**Published:** 2019-11-05

**Authors:** James Brooks, Daniele Faccio

**Affiliations:** School of Physics and Astronomy, University of Glasgow, Glasgow G12 8QQ, UK; james.alex.brooks@gmail.com

**Keywords:** LiDAR, non-line-of-sight, time-of-flight

## Abstract

The ability to locate a target around a corner is crucial in situations where it is impractical or unsafe to physically move around the obstruction. However, current techniques are limited to long acquisition times as they rely on single-photon counting for precise arrival time measurements. Here, we demonstrate a single-shot non-line-of-sight range-finding method operating at 10 Hz and capable of detecting a moving human target up to distances of 3 m around a corner. Due to the potential data acquisition speeds, this technique will find applications in search and rescue and autonomous vehicles.

## 1. Introduction

There has been significant interest in visualising and locating hidden objects in recent years [1,2,3,4,5,6,7,8,9,10,11,12,13,14,15,16,17,18,19,20,21]. In particular, the use of single photon avalanche detectors (SPADs) with laser-illuminated detection and ranging (LiDAR) techniques has allowed the 3D reconstruction of hidden objects [11,12,13,14] and tracking of a hidden target’s movement [16,18,19]. Both of these implementations take advantage of the high time resolution of SPADs and time-of-flight sensing techniques [11,12,16,18,19].

The general application of LiDAR in a non-line-of-sight (NLOS) situation is to send laser pulses toward the target via an intermediary surface and detect the backscattered light [18]. Due to SPADs being designed to operate beyond the breakdown voltage [11,22], they will only ever register one photon per pulse [22]. With a measurement requiring millions of detected photons to build up, lasers capable of very high repetition rates (10 MHz or more) ) are required to keep the acquisition time around 1 s. In the case of moving targets, however, even 1 s can be too long; ideally, a measurement would be taken on a single-shot basis.

In this work, we use an avalanche photodiode (APD) and a Q-switched laser to detect and send (respectively) more photons per pulse. We demonstrate detection of a moving target up to ∼ 2.5 m around a corner, which is comparable to the performance of SPAD-based systems [18]. We then show the feasibility of single-shot measurements in the form of a NLOS range-finder that can be modified to perform full target location.

## 2. Method

### 2.1. Experimental Setup

We utilise the general method used in previous work [16,18,19] of directing laser pulses towards a wall and observing the signal returned from an area on the same wall. For these experiments, the wall is a large piece of card.

The transceiver used in this work comprises an electro-optically Q-switched Nd:YAG laser (Quantum Light Instruments, Vilnius, Lithuania, Quantas Q-SPARK) with 1064 nm peak wavelength, 10 Hz repetition rate, ∼ 2 ns pulse duration, and 10 mJ pulse energy attenuated to ∼ 1 mJ and an InGaAs APD (Laser Components, Olching, Germany, A-CUBE-I200-240, 240 MHz bandwidth). An oscilloscope (Teledyne LeCroy, New York, NY, USA, WaveRunner 610Zi) is used to digitise the signals from measurement runs and data processing is currently done on a desktop afterwards; however, we note that, with the appropriate choice of hardware, there is no reason this processing cannot be done live.

**Figure 1:**
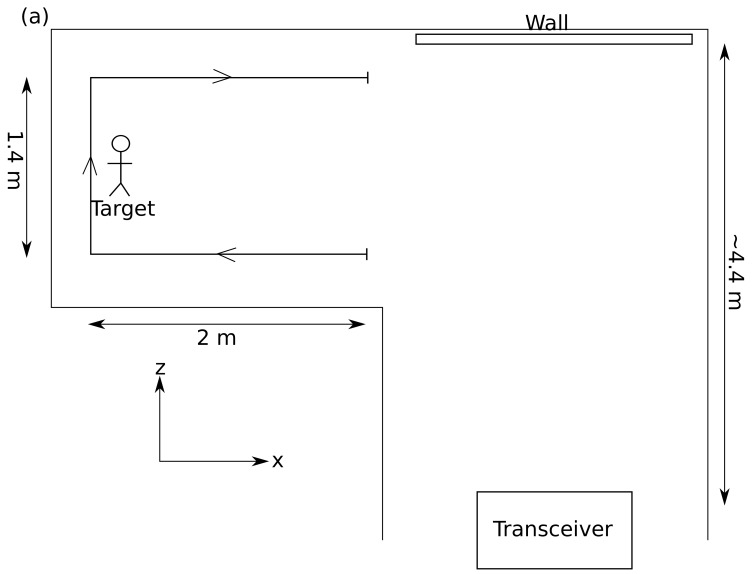

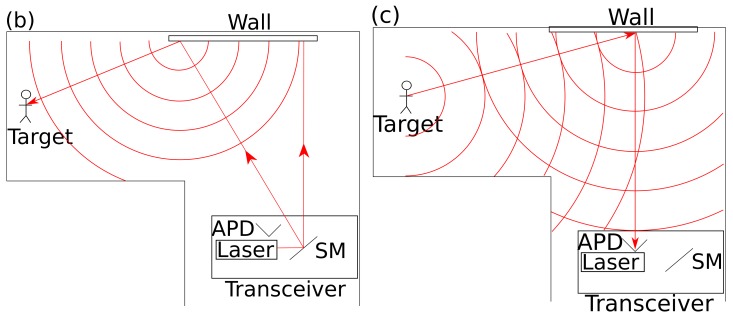
(**a**) Schematic (not-to-scale) representation of the setup used for this experiment. The wall is a cardboard screen covered with white paper. The target moves out of the direct line-of-sight along the path indicated. (**b**) Light path from transceiver to target. (**c**) Light path from target to APD. SM, scanning mirror.

We send a laser pulse from the transceiver on to the wall located ∼ 4.4 m away, as illustrated in Figure 1. The pulse hits the wall and scatters into an approximately spherical wavefront that propagates in all directions. Some of this light hits the target (a person in a white boiler suit) and is scattered back towards the wall. An area on the wall with diameter ∼ 4 cm is imaged onto the APD by a 2.5 cm diameter, 75 mm focal length lens. The signal recorded by the APD is saved and a scanning galvanometer mirror diverts the next pulse to a second spot on the wall. Whilst clothing reflectivity can change the return signal amplitude, we have found the dominant effect is the rapid decay of the signal with target distance from the scattering wall. This seems to be currently posing a strong limit on all measurements of the kind shown here (cf. [18]), limiting object distances to be of the order of 3 m from the scattering wall.

### 2.2. Position Retrieval

**Figure 2:**
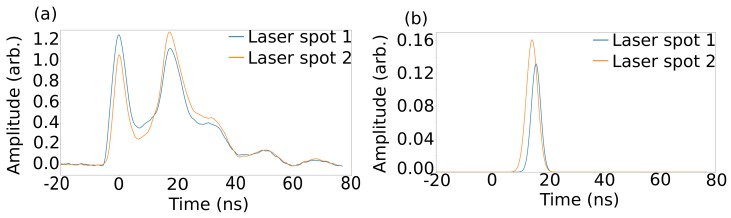
(**a**) Example signals recorded by the system after smoothing, one for each laser spot. (**b**) The Gaussian fits to the same signals after background subtraction.

The recorded signals are smoothed using a Savitzky–Golay filter (examples shown in Figure 2). We use the position of the first peak in the recorded signal (which is due to the first direct return scatter) as the “start” time for each waveform and define the timing of subsequent events relative to this. We then subtract a background signal taken as the median amplitude at each time over all frames with the same laser spot used, as in [16]. This removes all signals from static objects and leaves the signal from the target as the only thing moving in this experiment.

We then locate the position of the most prominent peak in the background-subtracted signal, which corresponds to the target, for laser position *i* and fit a Gaussian to it (examples shown in Figure 2). To retrieve the target position, we utilise the method described in [16,18,19]. In summary, we define a probability distribution for each laser position on the wall as:

(1) $$P_{i}\left(\vec{r_{0}}\right)\propto \exp\left[-\frac{\left(\right|\vec{r_{0}}-\vec{r_{i}}\left|+\right|\vec{r_{0}}-\vec{r_{d}}{\left|-ct_{i}\right)}^{2}}{2\sigma^{2}}\right]$$

where *c* is the speed of light, $\vec{r_{i}}=\left(x_{i},y_{i},z_{i}\right)$ is the laser position on the wall, $\vec{r_{0}}=\left(x_{0},y_{0},z_{0}\right)$ is the target position, $\vec{r_{d}}=\left(x_{d},y_{d},z_{d}\right)$ is the position of the centre of the observation area and $t_{i}$ is the time of flight. σ is the uncertainty in the time of flight measurement and is taken to be the standard deviation from the Gaussian fit. To locate the target, we multiply the probability distributions from the two laser spots which overlap at the target position. We underline that we are working under the assumption that the hidden target is, e.g., a person and therefore we restrict the problem to searching for the 2D location or even just simple distance from the wall (as opposed to searching for the full 3D location.

## 3. Results

### 3.1. 2D Localisation

We start by replicating the experiment in [18]. In our experiment, the target moves at a slow (∼ 25 cm·s${}^{-1}$) walk in a “C” shape around the corner, travelling 2 m parallel to the wall and 1.4 m perpendicular to the wall (shown schematically in Figure 1). At this speed, the distance moved by the target between the two measurements required for location is significantly smaller than the expected distance resolution of the system given by the pulse duration (∼ 60 cm); therefore, we make the approximation that the target does not move between laser pulses. The laser spots are located at $\vec{r_{1}}$ = ( 34 cm, −45 cm, 0 cm) and $\vec{r_{2}}$ = (−83 cm, −54 cm, 0 cm) with the observation point chosen as the origin of the coordinate system. To simplify the retrieval algorithm, we restrict the system to looking at the object at one height above the floor ($y_{0}=0\;\mathrm{cm}$) and search for the target in the (x,z) plane (see Figure 1).

**Figure 3:**
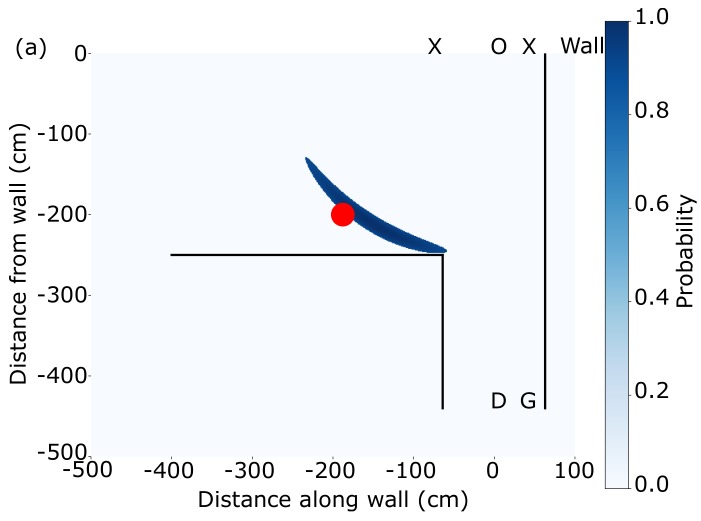

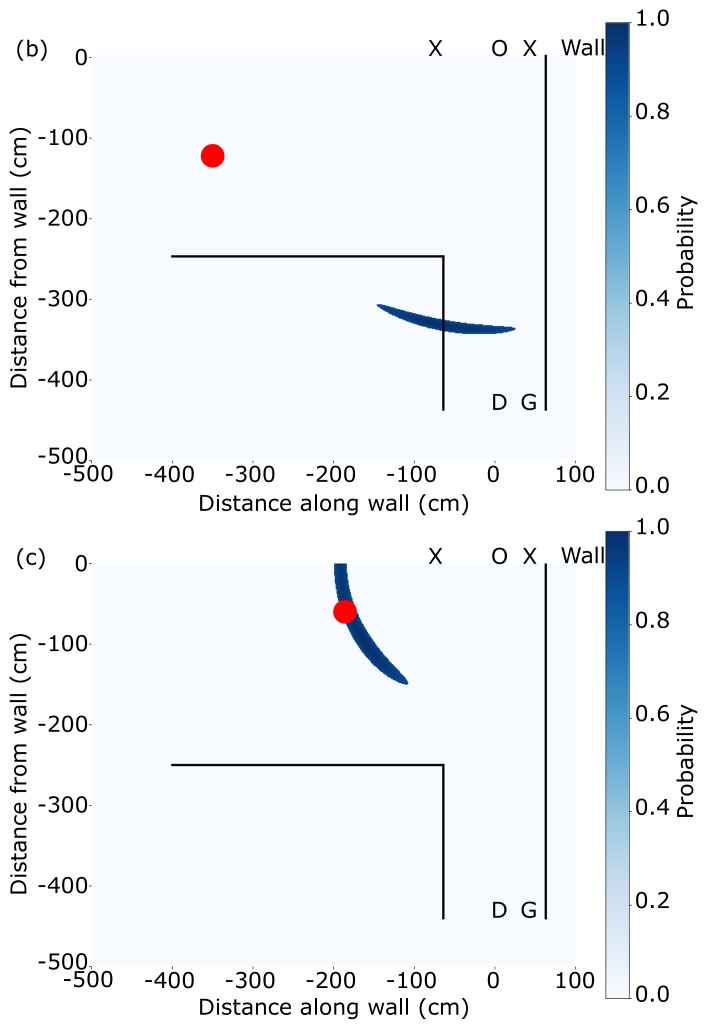
Highest probability position (>90%, blue) for three target positions in (**a**–**c**) (target position in the three figures is shown as a red circle) showing that the system can locate a hidden target up to around 2.5 m around a corner. Black lines denote walls, D is the APD position, G is the scanning galvanometer mirror position, O is the observation point and the Xs show the laser spot positions used.

Figure 3 shows the retrieved target location for different actual positions after calibration. The blue regions correspond to probabilities greater than 90% and the red circles show the true position. We note that, due to the poorer time-resolution of APDs compared to SPADs and the longer pulse duration of the laser, our regions of highest probability are broader than previously shown with SPAD-based systems [18]. This could be improved by using shorter pulse durations and/or an APD with a greater bandwidth. Figure 3 shows measurements taken at different times, 1.4s, 10.6s and 19.8s from the acquisition start time. As can be seen, the target is correctly located when it is at distances $\vec{r_{0}}≲2.5\;m$. At greater distances, the return signal is too small to be seen due to its $\frac{1}{d^{6}}$ dependence on distance. We underline that each measurement is the result of just two laser shot acquisitions and therefore takes 0.2 s.

### 3.2. Single-Shot Range-Finding

We now move to demonstrate a single-shot range-finding system. To do this, we use the data recorded above from only the laser spot closest to the observation point ( 34 cm to the right of the observation point). We measure the time of flight (*t*) as before but this time we assume that the distance to the target is $|\vec{r_{0}}|\approx \frac{ct}{2}$. This has the added benefit of simplifying the algorithm used as no probability ellipses need to be calculated, allowing faster processing. However, it does only retrieve the distance of the target from the wall rather than its absolute position in (x,z) coordinates. We note that this distance information is typically sufficient when there is contextual information from the scene itself to allow location of the target. Usually, a simple image or observation of the scene will allow the immediate location of the target. For example, an image of the scene indicating that the hidden environment is a corridor or is constrained to a limited volume by walls, will immediately rule out a very wide range of options; effectively restricting the target position to within the measurement uncertainty obtained even with two or multiple laser pulse measurements.

**Figure 4:**
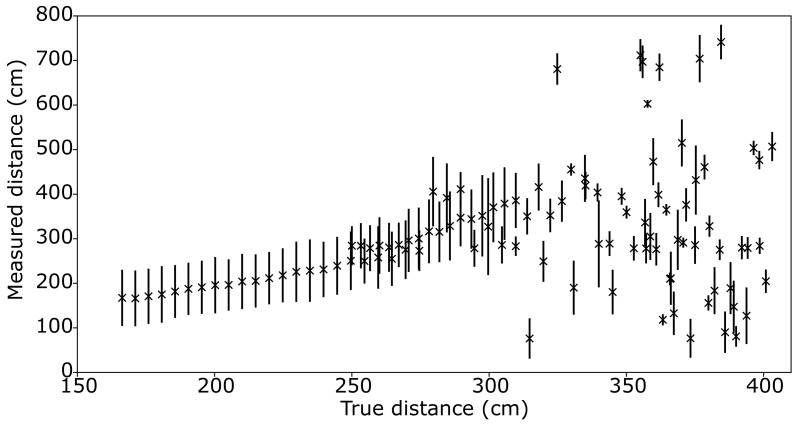
Distance to hidden target measured by range-finder against the ground truth after calibration showing that the system returns the correct range up to true distances around 3 m. The straight line fit to true distances <2.5 m returned a gradient of 0.98.

Figure 4 shows the measured distance to target against the true distance. In Figure 4, it is clear that the system works quite well up to distances around 3 m. This is similar to distance demonstrated with other systems based on single-photon sensitive detectors and millions of femtosecond laser pulse measurements, whilst, here, these results are obtained with a single, nanosecond laser shot and an off-the-shelf APD detector. Due to this, measurements can be performed at the laser repetition rate of 10 Hz, 10× faster than previous SPAD-based demonstrations.

## 4. Conclusions

We have shown that a moving target hidden from the direct line-of-sight can be located using an APD up to a similar range as reported with SPADs [18]. Furthermore, using the APD allows for shorter acquisition times as measurements can be taken at the laser repetition rate of 10 Hz. A single-shot non-line-of-sight range-finder has been demonstrated with a maximum range of 3 m. We note that non-line-of-sight imaging can be rendered relatively insensitive to ambient of sunlight illumination by placing narrow-bandpass filters in front of the detector so as to filter out all light except for the laser [18]. Moreover, moving towards infrared wavelengths provides an additional advantage due to lower sunlight levels (see, e.g., [23] for photon budget calculations proving that indeed sunlight illumination can be suppressed so that the return laser signal dominates the final measurement). The single-shot measurement could be extended to 2D target location by either using multiple detectors or splitting the laser pulse into two, directing one through a delay line and separating the return signals in software. Thus, the number of measurements per second will only be limited by the laser repetition rate and/or the speed of the acquisition and processing hardware.

The scope of this work is to demonstrate a proof-of-concept approach to non-line-of-sight detection that can identify the presence and also position of a hidden object and that uses simple, inexpensive and off-the-shelf components yet still achieving the fastest detection rates shown to date. Further work would be required to fully identify components and specifications of a system that could be deployed by a vehicle or potentially even a hand-held device for rescue and military missions.
